# Supplementary material for: Usefulness of Discriminant Analysis in the Morphometric Differentiation of Six Native Freshwater Species from Ecuador
Source: Animals (Basel). 2021 Jan 7;11(1):111. doi: 10.3390/ani11010111 (PMC7827686; doi:10.3390/ani11010111)
Supplement: Supplementary file 1 [file animals-11-00111-s001.pdf]

animals

Supplementary Material

Usefulness of Discriminant Analysis in the Morphometric Differentiation of Six Native Freshwater Species from Ecuador

Ana Gonzalez-Martinez [1], Carmen De-Pablos-Heredero [2], Martin González [3], Jorge Rodriguez [3], Cecilio Barba [1] and Antón García [1,*]

[1] Department of Animal Production, Faculty of Veterinary Sciences, University of Cordoba, 14071 Córdoba, Spain; agmartinez@uco.es (A.G.-M.); cjbarba@uco.es (C.B.)
[2] Department of Business Economics (Administration, Management and Organization), Applied Economics II and Fundamentals of Economic Analysis, ESIC Business & Marketing School, Rey Juan Carlos University, Paseo de los Artilleros s/n, 28032 Madrid, Spain; carmen.depablos@urjc.es
[3] Department of Animal Production, Quevedo State Technical University, Av. Quito km. 1 1/2 vía a Santo Domingo de los Tsáchilas. Quevedo, 120501 Los Ríos, Ecuador; mgonzalez@uteq.edu.ec (M.G.); jrodriguez@uteq.edu.ec (J.R.)
* Correspondence: pa1gamaa@uco.es

Citation: Gonzalez-Martinez, A.; De-Pablos-Heredero, C.; González, M.; Rodriguez, J.; Barba, C.; García, A. Usefulness of discriminant analysis in the morphometric differentiation of six native freshwater species from Ecuador. *Animals* **2021**, *11*, 111. https://doi.org/10.3390/ani11010111

Received: 29 November 2020
Accepted: 5 January 2021
Published: 7 January 2021

Publisher's Note: MDPI stays neutral with regard to jurisdictional claims in published maps and institutional affiliations.

Copyright: © 2021 by the authors. Submitted for possible open access publication under the terms and conditions of the Creative Commons Attribution (CC BY) license (http://creativecommons.org/licenses/by/4.0/).

Table S1. Descriptive statistics of the ratio between body measurements and standard length (original data) of six freshwater species from Ecuador (Mean ± Standard error (Coeffcient of Variation)).

**Character** [1]	*Cichlasoma festae*	*Andinoacara rivulatus*	*Dormitator latifrons*	*Brycon dentex*	*Hoplias microlepis*	*Leporinus ecuadorensis*	**ANOVA** [2]
BW/SL	6.22 ^{a} ± 0.16 (18.01)	10.80 ^{c} ± 0.15 (14.43)	8.84^{b} ± 0.21 (23.81)	9.72 ^{bc} ± 0.33 (49.85)	13.48 ^{d} ± 0.73 (55.72)	5.60 ^{a} ± 0.17 (32.44)	45.93 ***
TL/SL	1.29 ^{a} ± 0.01 (7.95)	1.28 ^{a} ± 0.00 (3.81)	1.28 ^{a} ± 0.00 (1.64)	1.37 ^{d} ± 0.01 (6.59)	1.18 ^{c} ± 0.01 (6.94)	1.10 ^{b} ± 0.00 (2.32)	251.80 ***
HL/SL	0.38 ^{e} ± 0.01 (14.21)	0.34 ^{a} ± 0.00 (7.81)	0.34 ^{a} ± 0.00 (7.69)	0.36 ^{d} ± 0.00 (9.11)	0.26 ^{c} ± 0.00 (16.28)	0.21 ^{b} ± 0.00 (7.85)	436.62 ***
ED/SL	0.09 ^{b} ± 0.00 (13.87)	0.08 ^{c} ± 0.00 (17.50)	0.05 ^{a} ± 0.00 (15.62)	0.08 ^{b} ± 0.00 (21.89)	0.04 ^{a} ± 0.00 (20.00)	0.04 ^{a} ± 0.00 (18.76)	300.19 ***
Pre-OL/SL	0.16 ^{b} ± 0.01 (26.19)	0.15 ^{b} ± 0.00 (15.69)	0.06 ^{d} ± 0.00 (17.58)	0.08 ^{a} ± 0.00 (16.67)	0.04 ^{c} ± 0.00 (31.42)	0.07 ^{a} ± 0.00 (14.08)	599.69 ***
Pre-DL/SL	0.38 ^{a} ± 0.01 (13.48)	0.44 ^{b} ± 0.00 (6.94)	0.40 ^{a} ± 0.00 (4.25)	0.82 ^{d} ± 0.00 (8.04)	0.51 ^{c} ± 0.00 (9.19)	0.44 ^{b} ± 0.00 (4.84)	2,055.92 ***
Pre-PcL/SL	0.40 ^{ab} ± 0.01 (10.90)	0.40 ^{b} ± 0.00 (7.25)	0.34 ^{e} ± 0.00 (6.65)	0.38 ^{a} ± 0.00 (14.77)	0.27 ^{d} ± 0.01 (26.35)	0.23 ^{c} ± 0.00 (5.98)	246.22 ***
Pre-PvL/SL	0.41 ^{ab} ± 0.01 (11.43)	0.44 ^{bc} ± 0.00 (6.61)	0.18 ^{d} ± 0.01 (38.14)	0.73 ^{e} ± 0.01 (20.44)	0.36 ^{a} ± 0.01 (26.10)	0.47 ^{c} ± 0.01 (30.67)	393.18 ***
Pre-AL/SL	0.65 ^{a} ± 0.01 (13.46)	0.73 ^{b} ± 0.00 (6.63)	0.67 ^{a} ± 0.00 (4.76)	1.01 ^{d} ± 0.01 (20.08)	0.79 ^{c} ± 0.01 (10.91)	0.69 ^{ab} ± 0.00 (3.67)	179.47 ***
DFL/SL	0.42 ^{d} ± 0.01 (11.31)	0.61 ^{e} ± 0.00 (5.87)	0.16 ^{a} ± 0.00 (8.48)	0.16 ^{a} ± 0.00 (21.47)	0.11 ^{b} ± 0.00 (25.50)	0.13 ^{c} ± 0.00 (8.58)	4,544.64 ***
DFRL/SL	0.41 ^{e} ± 0.01 (10.16)	0.07 ^{b} ± 0.00 (40.54)	0.12 ^{a} ± 0.00 (12.51)	0.23 ^{d} ± 0.00 (22.14)	0.13 ^{a} ± 0.00 (31.58)	0.19 ^{c} ± 0.00 (11.20)	728.72 ***
PcFL/SL	0.57 ^{f} ± 0.01 (16.16)	0.32 ^{e} ± 0.00 (10.32)	0.23 ^{c} ± 0.00 (7.64)	0.28 ^{d} ± 0.00 (13.94)	0.09 ^{a} ± 0.00 (32.78)	0.12 ^{b} ± 0.00 (16.95)	1,391.334***
PvFL/SL	0.45 ^{f} ± 0.01 (10.26)	0.32 ^{e} ± 0.00 (11.26)	0.21 ^{d} ± 0.00 (10.62)	0.19 ^{c} ± 0.00 (21.00)	0.12 ^{a} ± 0.00 (27.08)	0.15 ^{b} ± 0.00 (9.03)	1,025.63 ***
AFL/SL	0.33 ^{d} ± 0.01 (30.82)	0.22 ^{c} ± 0.00 (8.83)	0.14 ^{b} ± 0.00 (15.35)	0.37 ^{e} ± 0.01 (27.08)	0.09 ^{a} ± 0.00 (22.12)	0.10 ^{a} ± 0.00 (12.66)	454.19 ***
AFRL/SL	0.23 ^{b} ± 0.00 (14.86)	0.24 ^{b} ± 0.00 (18.77)	0.11 ^{a} ± 0.00 (29.76)	0.16 ^{d} ± 0.00 (26.25)	0.09 ^{a} ± 0.00 (29.30)	0.13 ^{c} ± 0.00 (9.98)	293.74 ***
UJL/SL	0.07 ^{bc} ± 0.00 (17.22)	0.04 ^{a} ± 0.00 (19.06)	0.06 ^{d} ± 0.00 (17.46)	0.07 ^{b} ± 0.00 (24.02)	0.07 ^{c} ± 0.00 (28.16)	0.04 ^{a} ± 0.00 (17.57)	82.88 ***
AC1/SL	0.39 ^{a} ± 0.01 (10.94)	0.54 ^{e} ± 0.01 (17.46)	0.33 ^{d} ± 0.00 (9.51)	0.39 ^{a} ± 0.01 (18.96)	0.12 ^{b} ± 0.00 (27.58)	0.23 ^{c} ± 0.01 (30.90)	534.17 ***
AC2/SL	0.35 ^{b} ± 0.01 (12.43)	0.49 ^{d} ± 0.01 (20.21)	0.28 ^{a} ± 0.00 (7.30)	0.36 ^{b} ± 0.00 (20.10)	0.16 ^{c} ± 0.00 (27.08)	0.27 ^{a} ± 0.01 (29.10)	298.56 ***
AC3/SL	0.14 ^{ab} ± 0.00 (13.15)	0.18 ^{d} ± 0.00 (15.58)	0.15 ^{abc} ± 0.00 (5.64)	0.16 ^{bc} ± 0.01 (70.20)	0.18 ^{cd} ± 0.00 (26.77)	0.13 ^{a} ± 0.00 (25.20)	10.09 ***
P1/SL	0.94 ^{a} ± 0.02 (11.58)	1.14 ^{e} ± 0.01 (9.48)	0.70 ^{d} ± 0.01 (9.72)	0.89 ^{a} ± 0.02 (24.86)	0.45 ^{b} ± 0.01 (29.79)	0.57 ^{c} ± 0.01 (13.36)	301.02 ***
P2/SL	0.80 ^{ab} ± 0.01 (11.12)	1.05 ^{c} ± 0.01 (10.21)	0.64 ^{a} ± 0.01 (10.23)	0.93 ^{bc} ± 0.05 (77.35)	0.46 ^{d} ± 0.01 (31.91)	0.64 ^{a} ± 0.01 (13.34)	32.02 ***
P3/SL	0.33 ^{a} ± 0.00 (12.15)	0.42 ^{d} ± 0.00 (9.91)	0.36 ^{ab} ± 0.00 (11.50)	0.38 ^{bc} ± 0.01 (21.63)	0.41 ^{cd} ± 0.01 (30.02)	0.48 ^{e} ± 0.01 (14.31)	8.77 ***
LC1/SL	0.16 ^{a} ± 0.00 (13.68)	0.18 ^{d} ± 0.00 (11.88)	0.17 ^{a} ± 0.00 (12.18)	0.17 ^{a} ± 0.00 (22.76)	0.10 ^{b} ± 0.00 (28.42)	0.11 ^{c} ± 0.00 (14.73)	159.43 ***
LC2/SL	0.11 ^{ab} ± 0.00 (28.74)	0.13 ^{c} ± 0.00 (13.71)	0.15 ^{d} ± 0.00 (8.62)	0.18 ^{e} ± 0.00 (26.23)	0.10 ^{a} ± 0.00 (27.95)	0.12 ^{bc} ± 0.00 (14.67)	110.85 ***
LC3/SL	0.05 ^{a} ± 0.00 (47.76)	0.06 ^{ab} ± 0.00 (18.22)	0.11 ^{d} ± 0.00 (10.39)	0.16 ^{e} ± 0.00 (26.53)	0.08 ^{c} ± 0.00 (33.98)	0.07 ^{b} ± 0.00 (15.73)	182.59 ***

[1] BW = body weight; SL = standard length; TL = total length; HL = head length; ED = eye diameter; Pre-OL = pre-orbital length; Pre-DL = pre-dorsal fin length; Pre-PcL = pre-pectoral fin length; Pre-PvL = pre-pelvic fin length; Pre-AL = pre-anal fin length; DFL = dorsal fin length; DFRL = dorsal fin ray length; PcFL = pectoral fin length; PvFL = pelvic fin length; AFL = anal fin length; AFRL = anal fin ray length; UJL = upper jaw length; AC1 = body depth 1; AC2 = body depth 2; AC3 = body depth 3; P1 = body perimeter 1; P2 = body perimeter 2; P3 = body perimeter 3; LC1 = body width 1; LC2 = body width 2; LC3 = body width 3. [2] *** $p < 0.001$. a, b, c, d, e superscript letters indicate significative differences between species ($p < 0.05$).

Table S2. Descriptive statistics of body measurements (adjusted data) of six freshwater species from Ecuador (Mean ± Standard Error (Coefficient of Variation)).

**Character** [1]	*Cichlasoma festae*	*Andinoacara rivulatus*	*Dormitator latifrons*	*Brycon dentex*	*Hoplias microlepis*	*Leporinus ecuad-orensis*	**ANOVA** [2]
BW	127.99 ^{a} ± 3.12 (17.22)	221.90 ^{c} ± 3.21 (14.76)	166.31 ^{d} ± 3.71 (22.28)	195.58 ^{b} ± 6.65 (49.77)	220.36 ^{bc} ± 11.40 (53.51)	106.65 ^{a} ± 3.32 (32.31)	39.82 ***
K	1.88 ^{a} ± 0.05 (17.22)	3.26 ^{c} ± 0.05 (14.76)	2.44 ^{d} ± 0.05 (22.28)	2.87 ^{b} ± 0.10 (49.77)	3.24 ^{bc} ± 0.17 (53.51)	1.57 ^{a} ± 0.05 (32.31)	39.82 ***
TL	23.34 ^{a} ± 0.23 (6.82)	23.34 ^{a} ± 0.08 (3.45)	24.33 ^{b} ± 0.05 (2.25)	25.27 ^{d} ± 0.12 (6.82)	24.07 ^{b} ± 0.16 (6.81)	20.88 ^{c} ± 0.05 (2.61)	167.93***
HL	6.37 ^{a} ± 0.10 (11.50)	5.80 ^{c} ± 0.04 (7.01)	6.39 ^{a} ± 0.06 (9.76)	6.32 ^{a} ± 0.04 (10.18)	6.04 ^{d} ± 0.08 (13.02)	3.88 ^{b} ± 0.04 (9.71)	277.94 ***
ED	1.28 ^{cd} ± 0.02 (10.03)	1.15 ^{b} ± 0.02 (15.44)	0.88 ^{a} ± 0.02 (17.34)	1.34 ^{d} ± 0.02 (21.28)	1.21 ^{bc} ± 0.03 (21.93)	0.79 ^{a} ± 0.02 (21.75)	119.68 ***
Pre-OL	2.16 ^{c} ± 0.08 (24.54)	2.09 ^{c} ± 0.03 (15.57)	1.21 ^{a} ± 0.02 (20.63)	1.17 ^{a} ± 0.02 (20.35)	1.38 ^{b} ± 0.04 (32.15)	1.41 ^{b} ± 0.03 (18.60)	175.30 ***
Pre-DL	6.73 ^{b} ± 0.12 (13.03)	7.89 ^{a} ± 0.05 (6.30)	7.58 ^{a} ± 0.04 (5.10)	14.90 ^{e} ± 0.08 (8.08)	10.64 ^{d} ± 0.09 (8.58)	8.33 ^{c} ± 0.05 (5.83)	1874.57 ***
Pre-PcL	6.47 ^{a} ± 0.07 (8.16)	6.51 ^{a} ± 0.04 (5.99)	6.39 ^{a} ± 0.05 (8.57)	6.45 ^{a} ± 0.07 (14.74)	6.41 ^{a} ± 0.12 (19.82)	4.38 ^{b} ± 0.04 (8.68)	119.89 ***
Pre-PvL	6.20 ^{a} ± 0.09 (9.69)	6.67 ^{a} ± 0.05 (8.38)	3.36 ^{b} ± 0.12 (35.29)	11.72 ^{e} ± 0.12 (15.41)	9.87 ^{d} ± 0.19 (19.41)	8.61 ^{c} ± 0.20 (24.02)	443.94 ***
Pre-AL	11.68 ^{b} ± 0.21 (12.73)	13.12 ^{a} ± 0.09 (6.61)	12.77 ^{a} ± 0.07 (5.46)	18.36 ^{d} ± 0.23 (18.53)	16.22 ^{c} ± 0.18 (11.31)	13.12 ^{a} ± 0.06 (4.98)	190.39 ***
DFL	5.72 ^{d} ± 0.12 (15.41)	8.45 ^{e} ± 0.09 (10.70)	3.09 ^{b} ± 0.05 (16.83)	2.46 ^{a} ± 0.04 (23.21)	3.86 ^{c} ± 0.09 (23.57)	2.47 ^{a} ± 0.04 (18.17)	1262.12 ***
DFRL	6.94 ^{f} ± 0.08 (7.71)	1.20 ^{a} ± 0.05 (40.14)	2.20 ^{b} ± 0.03 (13.93)	3.93 ^{e} ± 0.05 (20.15)	2.99 ^{c} ± 0.07 (24.35)	3.60 ^{d} ± 0.04 (10.45)	724.74 ***
PcFL	7.11 ^{e} ± 0.16 (16.32)	4.09 ^{a} ± 0.06 (15.88)	4.56 ^{d} ± 0.09 (20.55)	4.00 ^{a} ± 0.06 (22.99)	3.57 ^{c} ± 0.11 (33.19)	2.27 ^{b} ± 0.07 (32.12)	199.69 ***
PvFL	6.44 ^{e} ± 0.07 (8.14)	4.65 ^{d} ± 0.06 (13.01)	4.01 ^{c} ± 0.07 (17.67)	2.94 ^{a} ± 0.04 (17.86)	3.59 ^{b} ± 0.09 (25.36)	2.78 ^{a} ± 0.04 (15.54)	351.90 ***
AFL	4.17 ^{d} ± 0.17 (28.80)	2.81 ^{a} ± 0.03 (12.50)	2.77 ^{a} ± 0.06 (21.82)	5.27 ^{e} ± 0.09 (24.60)	3.59 ^{c} ± 0.11 (32.99)	1.83 ^{b} ± 0.05 (26.86)	225.51 ***
AFRL	3.37 ^{b} ± 0.07 (14.18)	3.53 ^{b} ± 0.06 (18.62)	2.03 ^{c} ± 0.07 (35.19)	2.55 ^{a} ± 0.04 (24.20)	2.81 ^{d} ± 0.07 (25.87)	2.37 ^{a} ± 0.03 (13.32)	83.79 ***
UJL	1.29 ^{a} ± 0.03 (17.61)	0.86 ^{b} ± 0.02 (19.14)	1.10 ^{c} ± 0.02 (17.74)	1.25 ^{a} ± 0.02 (24.17)	1.28 ^{a} ± 0.04 (29.15)	0.80 ^{b} ± 0.01 (17.63)	76.65 ***
AC1	4.73 ^{ab} ± 0.10 (14.96)	6.74 ^{c} ± 0.16 (23.62)	6.56 ^{c} ± 0.16 (23.65)	5.30 ^{d} ± 0.05 (14.42)	4.69 ^{b} ± 0.08 (18.16)	4.23 ^{a} ± 0.10 (25.00)	88.01 ***
AC2	4.71 ^{b} ± 0.07 (10.74)	6.75 ^{d} ± 0.16 (24.41)	5.37 ^{a} ± 0.09 (16.63)	5.26 ^{a} ± 0.05 (12.77)	5.19 ^{ac} ± 0.06 (12.64)	4.90 ^{bc} ± 0.11 (22.35)	53.01 ***
AC3	2.50 ^{ab} ± 0.05 (12.92)	3.36 ^{cd} ± 0.05 (15.59)	2.89 ^{ab} ± 0.02 (5.76)	2.88 ^{b} ± 0.14 (69.44)	3.53 ^{d} ± 0.08 (24.20)	2.43 ^{a} ± 0.06 (24.32)	12.71 ***
P1	13.04 ^{a} ± 0.17 (9.24)	15.99 ^{e} ± 0.10 (6.66)	14.86 ^{c} ± 0.21 (14.20)	13.43 ^{ab} ± 0.18 (19.60)	14.10 ^{bc} ± 0.19 (14.12)	10.62 ^{d} ± 0.13 (12.72)	87.58 ***
P2	11.37 ^{a} ± 0.11 (6.98)	14.98 ^{c} ± 0.11 (7.48)	12.23 ^{ab} ± 0.18 (14.59)	14.02 ^{bc} ± 0.62 (64.87)	14.20 ^{bc} ± 0.24 (17.68)	11.99 ^{a} ± 0.13 (11.33)	6.91 ***
P3	5.96 ^{b} ± 0.09 (11.00)	7.60 ^{c} ± 0.07 (8.90)	6.75 ^{a} ± 0.07 (10.32)	6.96 ^{a} ± 0.09 (19.83)	8.34 ^{d} ± 0.20 (25.44)	9.03 ^{e} ± 0.11 (13.29)	13.82 ***
LC1	2.48 ^{c} ± 0.04 (12.84)	2.80 ^{a} ± 0.02 (8.98)	3.27 ^{d} ± 0.05 (15.64)	2.77 ^{a} ± 0.03 (17.76)	2.75 ^{a} ± 0.04 (14.21)	2.12 ^{b} ± 0.03 (12.93)	87.42 ***
LC2	1.77 ^{c} ± 0.07 (28.26)	2.08 ^{d} ± 0.02 (12.08)	2.86 ^{b} ± 0.03 (10.10)	2.98 ^{b} ± 0.05 (22.42)	2.50 ^{a} ± 0.04 (17.28)	2.32 ^{a} ± 0.03 (12.64)	97.93 ***
LC3	0.89 ^{a} ± 0.05 (41.99)	1.10 ^{a} ± 0.02 (17.92)	2.08 ^{d} ± 0.02 (11.13)	2.83 ^{e} ± 0.05 (24.02)	1.85 ^{c} ± 0.05 (26.43)	1.34 ^{b} ± 0.02 (13.93)	194.98 ***

[1] BW = body weight; K = Fulton´s factor; TL = total length; HL = head length; ED = eye diameter; Pre-OL = pre-orbital length; Pre-DL = pre-dorsal fin length; Pre-PcL = pre-pectoral fin length; Pre-PvL = pre-pelvic fin length; Pre-AL = pre-anal fin length; DFL = dorsal fin length; DFRL = dorsal fin ray length; PcFL = pectoral fin length; PvFL = pelvic fin length; AFL = anal fin length; AFRL = anal fin ray length; UJL = upper jaw length; AC1 = body depth 1; AC2 = body depth 2; AC3 = body depth 3; P1 = body perimeter 1; P2 = body perimeter 2; P3 = body perimeter 3; LC1 = body width 1; LC2 = body width 2; LC3 = body width 3. [2] *** $p < 0.001$. a, b, c, d superscript letters indicate significative differences between species ($p < 0.05$).
